# GFAP point-of-care measurement for prehospital diagnosis of intracranial hemorrhage in acute coma

**DOI:** 10.1186/s13054-024-04892-5

**Published:** 2024-04-05

**Authors:** Sabina Zylyftari, Sebastian Luger, Kristaps Blums, Stephan Barthelmes, Sebastian Humm, Hannsjörg Baum, Stephan Meckel, Jörg Braun, Gregor Lichy, Andreas Heilgeist, Love-Preet Kalra, Christian Foerch

**Affiliations:** 1 Department of Neurology, RKH Klinikum Ludwigsburg, Posilipostr. 4, 71640 Ludwigsburg, Germany; 2 Department of Neurology, University Hospital Frankfurt, Goethe-University, Frankfurt am Main, Germany; 3 Institute for Laboratory Medicine and Transfusion Medicine, RKH Regionale Kliniken Holding Und Services GmbH, Ludwigsburg, Germany; 4 Institute of Diagnostic and Interventional Neuroradiology, RKH Klinikum, Ludwigsburg, Germany; 5 Department of Anesthesiology, Intensive Care Medicine and Pain Medicine, RKH Klinikum, Ludwigsburg, Germany; 6 DRF Luftrettung, Stuttgart, Germany

**Keywords:** GFAP, Glial fibrillary acidic protein, Biomarker, Diagnostics, Coma

## Abstract

**Background:**

Prehospital triage and treatment of patients with acute coma is challenging for rescue services, as the underlying pathological conditions are highly heterogenous. Recently, glial fibrillary acidic protein (GFAP) has been identified as a biomarker of intracranial hemorrhage. The aim of this prospective study was to test whether prehospital GFAP measurements on a point-of-care device have the potential to rapidly differentiate intracranial hemorrhage from other causes of acute coma.

**Methods:**

This study was conducted at the RKH Klinikum Ludwigsburg, a tertiary care hospital in the northern vicinity of Stuttgart, Germany. Patients who were admitted to the emergency department with the prehospital diagnosis of acute coma (Glasgow Coma Scale scores between 3 and 8) were enrolled prospectively. Blood samples were collected in the prehospital phase. Plasma GFAP measurements were performed on the i-STAT Alinity® (Abbott) device (duration of analysis 15 min) shortly after hospital admission.

**Results:**

143 patients were enrolled (mean age 65 ± 20 years, 42.7% female). GFAP plasma concentrations were strongly elevated in patients with intracranial hemorrhage (*n* = 51) compared to all other coma etiologies (3352 pg/mL [IQR 613–10001] vs. 43 pg/mL [IQR 29–91.25], *p* < 0.001). When using an optimal cut-off value of 101 pg/mL, sensitivity for identifying intracranial hemorrhage was 94.1% (specificity 78.9%, positive predictive value 71.6%, negative predictive value 95.9%). In-hospital mortality risk was associated with prehospital GFAP values.

**Conclusion:**

Increased GFAP plasma concentrations in patients with acute coma identify intracranial hemorrhage with high diagnostic accuracy. Prehospital GFAP measurements on a point-of-care platform allow rapid stratification according to the underlying cause of coma by rescue services. This could have major impact on triage and management of these critically ill patients.

**Graphical Abstract:**

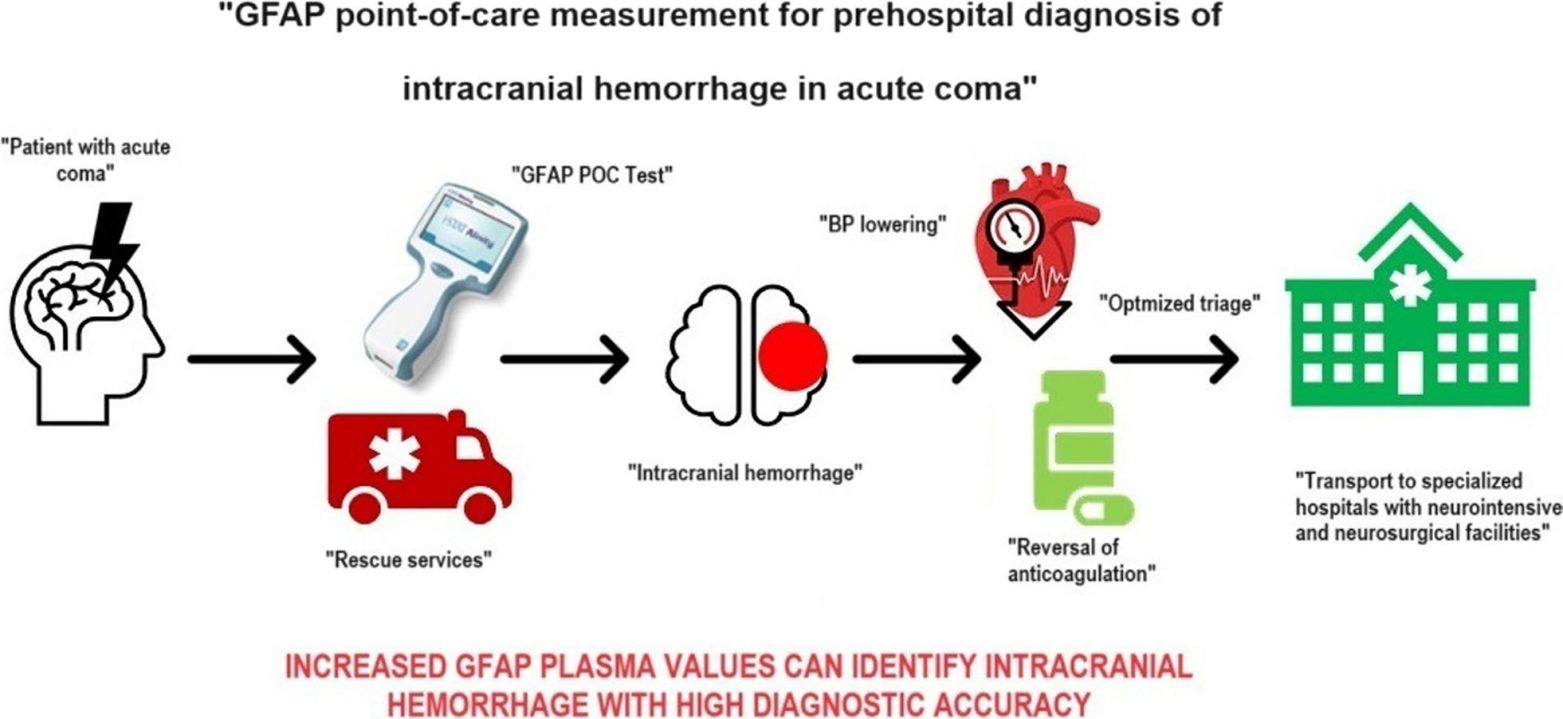

## Background

The underlying etiology in patients with acute coma is highly diverse, and prehospital differentiation between entities is usually not possible. This puts utmost challenge on the management and triage of patients by rescue services, particularly in remote areas [1–3]. Patients with intracranial hemorrhage may require emergent transport to specialized hospitals with neuro-intensive and neurosurgical facilities, in order to limit sequelae of space-occupying hematoma expansion. In contrast, patients having primarily non-cerebral causes of acute coma such as pulmonary embolism, cardiac arrest, metabolic and hormonal derangement, or intoxication require prompt diagnostic measures and therapy in nearby internal medicine facilities [4, 5]. Thus, a widely applicable, easy-to-use and cost-effective stratification tool for the preclinical setting to differentiate intracranial hemorrhage from primarily non-cerebral causes of acute coma is urgently needed.

A highly promising biomarker in this context is glial fibrillary acidic protein (GFAP), a brain-specific cytoskeletal protein mainly expressed in astrocytes. Blood levels are low in healthy individuals as well as in patients with neurological conditions that do not involve glial damage. In intracerebral hemorrhage, however, GFAP is rapidly released from destructed tissue into the blood stream [6–10]. Similarly, for traumatic brain injury (TBI), studies showed that higher GFAP serum concentrations indicated “focal cerebral mass lesions” (i.e. intracerebral hemorrhage) [11–13].

All previous GFAP studies have been performed on stationary laboratory platforms. Until recently, a GFAP point-of-care test was not available [6–10]. However, in 2022, the U.S. Food and Drug Administration (FDA) approved the portable i-STAT Alinity® TBI plasma test (Abbott™) with the clinical indication for patients who suffered mild TBI to avoid unnecessary head CT scans [14]. This point-of-care test measures GFAP and the neuronal protein Ubiquitin C-terminal hydrolase L1 (UCH-L1) concentrations in plasma within 15 min.

The aim of this study was to test the diagnostic value of a GFAP point-of-care device to rapidly differentiate intracranial hemorrhage from other causes of acute coma in the prehospital phase.

## Methods

### Study design

This exploratory study was part of an ongoing biomarker project focusing on the identification of intracranial hemorrhage in the prehospital phase by measuring plasma GFAP on a point-of-care device. The guidelines of the Standards for Reporting of Diagnostic Accuracy (STARD) initiative were followed [15].

This study was conducted at the RKH Klinikum Ludwigsburg, a tertiary care hospital in the northern vicinity of Stuttgart, Baden Württemberg, Germany. The hospital provides acute neurological and neurosurgical service to approximately 550 000 inhabitants, with no other neurological or neurosurgical hospital around. All emergencies with an indication for hospital admission, including all patients with acute coma as the main clinical symptom are transported to this hospital by rescue services (by ground or by air). In addition, this hospital serves as a transregional trauma center, leading to admissions of trauma patients from a wide catchment area.

**Fig. 1:**
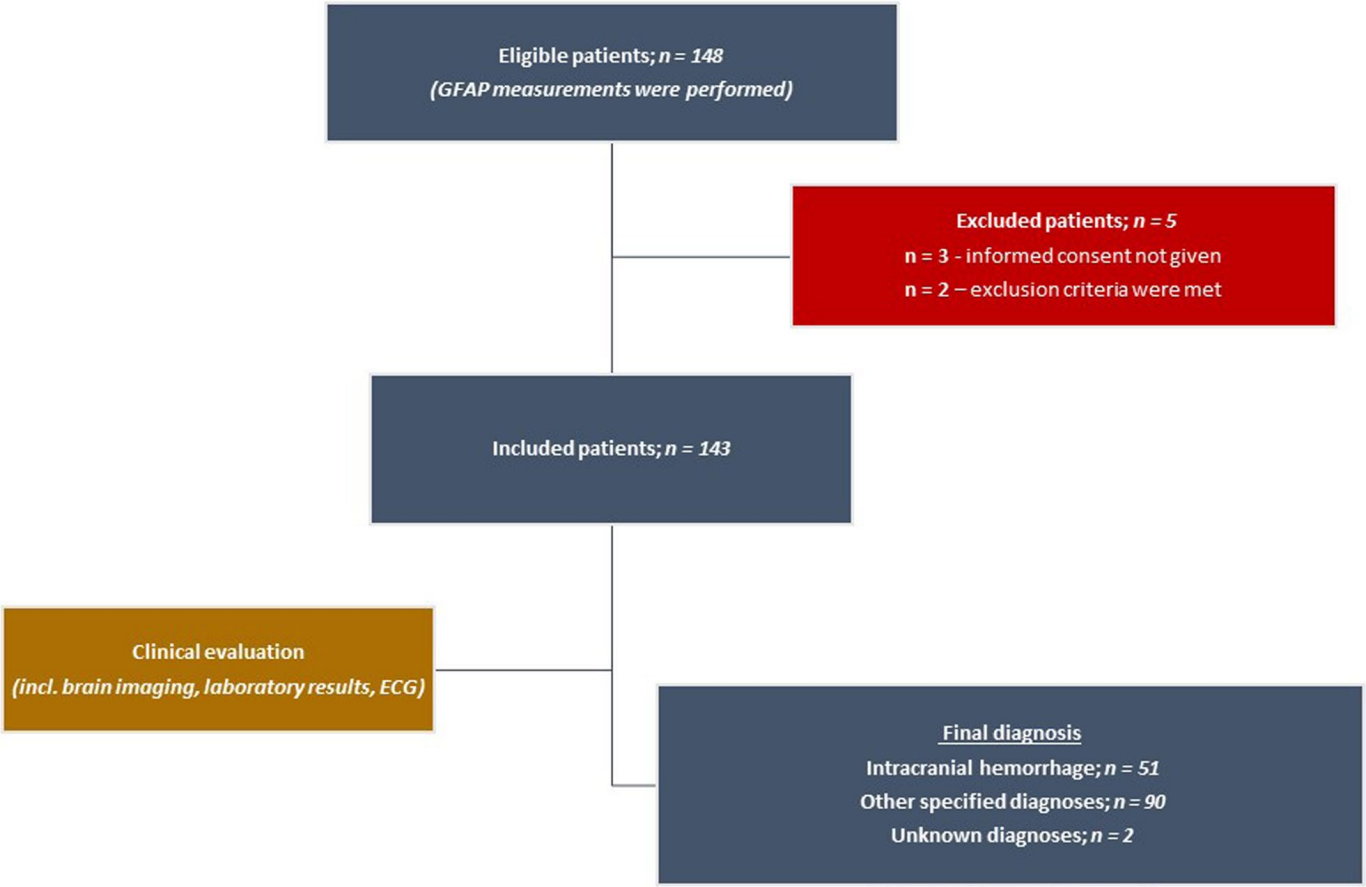
STARD flow diagram of the study

Between January 1st, 2023 and October 31st, 2023, patients who were admitted to the hospital with the prehospital diagnosis of acute coma and Glasgow Coma Scale score (GCS) between 3 and 8 were considered eligible for study inclusion. Patients younger than 18 years (if known) were not included. Once past medical history became available during in-hospital stay, the following exclusion criteria were applied: (I) ischemic stroke or transient ischemic attack in the past 3 months, (II) any intracranial hemorrhage in the past 3 months, (III) TBI in the past 3 months (current incident not included), and (IV) brain tumor at any time in the past medical history (data suggested that plasma GFAP is increased in patients with malignant glioma) (Fig. 1) [12, 16, 17].

The following clinical baseline variables were recorded: age, sex, first determined GCS, history of arterial hypertension, diabetes or hyperlipidemia (defined according to current guidelines), current and prior treatment with anticoagulants (vitamin K antagonists, direct oral anticoagulants) or antiplatelets, time interval between symptom onset and hospital admission (if known), resuscitation in the emergency setting, and trauma in the context of the emergency situation [18–20]. Immediate neurosurgical interventions and in-hospital mortality were also recorded.

The primary endpoint of the study was the underlying etiology of acute coma, categorized into intracranial hemorrhage (including intracerebral hemorrhage, subarachnoid hemorrhage, epidural hematoma and subdural hematoma) versus all other entities. The secondary endpoint categorized acute coma etiology into a primary cerebral cause (including intracranial hemorrhage as defined above, ischemic stroke, and CNS infection, but excluding epileptic seizures) versus all other entities. The diagnosis was established according to the International Classification of Diseases, 10th revision, on the basis of all available clinical data, brain imaging, laboratory results, and other examinations.

### Blood sampling and GFAP measurements

**Fig. 2:**
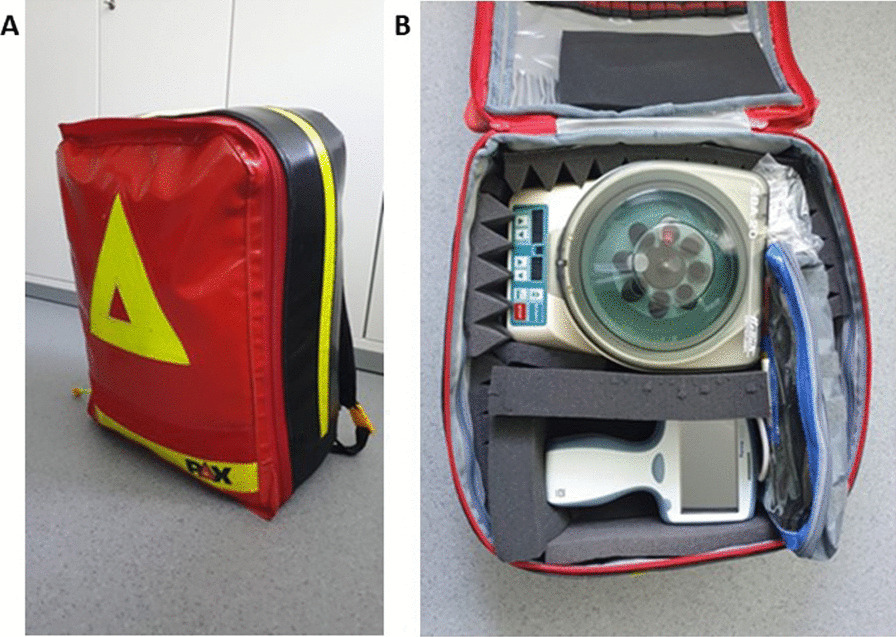
For the GFAP point-of-care measurements entirely performed in the prehospital phase (*n* = 5), a backpack was equipped. **A** Outer view of the PAX emergency backpack with the approximate dimensions 49 × 36 × 28 cm, weighing 8.0 kg. **B** Inside the backpack the i-STAT Alinity® device, a small portable centrifuge (visible) and a pipette with pipette consumables (not visible) are secured between foam

Blood samples for diagnostic purposes are routinely taken in the prehospital phase by rescue services according to local standard of practice protocols. GFAP measurements on the point-of-care platform were performed in the hospital immediately after the patient had arrived (together with the routine blood analysis). However, in 5 patients, we fully completed GFAP measurements in the prehospital phase (i.e. prior to hospital admission of the patient) in order to demonstrate feasibility of the process. For doing so, a backpack was equipped with a small centrifuge, the GFAP point-of-care device and supplemental material (such as pipettes) (Fig. 2). On selected dates in August 2023 (dependent on the availability of the study team), study personnel joined the rescue services if the emergency code contained the phrase “unconscious patient” (in German).

The i‐STAT® TBI plasma test consists of a single‐use test cartridge (i‐STAT® TBI plasma test cartridge) that functions with the i‐STAT Alinity® system, a portable in vitro diagnostic test. Immunoassays for GFAP and UCH‐L1 are evaluated simultaneously from a single plasma sample. Following centrifugation of the sample at 2000 g for 10 min, 20 µL of plasma was pipetted into the sample well of the i‐STAT® TBI plasma test cartridge, which was then inserted into the i‐STAT Alinity® system. Sample analysis took 15 min and concentrations of the two biomarkers were displayed on the analyzer screen. The reportable range for GFAP is 30–10,000 pg/mL, and the interrun coefficient of variation was shown to be less than 10% [21]. We substituted a saturated GFAP signal by the value of 10,001 pg/mL, which is 1 pg/mL above the highest measured concentration. We furthermore substituted GFAP concentrations displaying the lower limits on the device (i.e. < 30 pg/mL) by the value of 29 pg/mL.

### Statistical analysis

No data has been published so far that would allow for sample size calculation. Rather, a 10-month recruitment period with a total number of > 120 patients was targeted by the study team once the diagnostic potential of GFAP measurements in acute coma patients became noticeable in the context of the biomarker project mentioned above. Statistical analyses were performed using IBM ® SPSS ® Statistics, Version 29 (Statistical Package for the Social Sciences). Because GFAP plasma concentrations were not normally distributed between individuals, statistical comparisons were made using the nonparametric Mann–Whitney U test. The optimal plasma GFAP cut-off concentration to distinguish intracranial hemorrhage from other causes of acute coma was calculated using receiver operating characteristics (ROC)—curve analysis. Sensitivity and specificity measures as well as the positive predictive value (PPV) and the negative predictive value (NPV) were derived from cross-tabulations. A significance level of 0.05 was chosen for all tests.

## Results

### Baseline characteristics

GFAP plasma concentrations were determined in 148 patients initially considered eligible for study inclusion. Five patients were later on excluded (missing informed consent, *n* = 3; fulfilled exclusion criteria, *n* = 2; see flow chart in Fig. 1). Two patients were initially included with age below 18 years (age not known by rescue service) and 13 patients were included with GCS values above 8 (initially attributed as coma patients by rescue service). These patients were not excluded. Thus, the final analysis was based on 143 patients. Mean age was 65 ± 20 years and 42.7% of patients were female. Median GCS in the prehospital setting was 4 (IQR 3–6). 22.4% of patients underwent mechanical resuscitation in the prehospital phase, and 28.7% of patients had reported trauma in the context of the emergency situation. Anticoagulation intake was present in 21.7% of patients. Regarding the etiology of acute coma, 51 patients had intracranial hemorrhage as the primary cause (intracerebral hemorrhage, *n* = 24; subarachnoid hemorrhage, *n* = 22; subdural hematoma, *n* = 3; epidural hematoma, *n* = 2), and 90 patients had other etiologies (ischemic stroke, *n* = 21; epileptic seizure, *n* = 19; CNS infection, *n* = 1; cardio-pulmonary, *n* = 32; metabolic, *n* = 5; intoxication, *n* = 7; psychogenic, *n* = 5). Two patients were deceased prior to the diagnostic evaluation in the hospital. These patients were classified as “unknown etiology” and were excluded from predictive analyses (see below).

**Fig. 3:**
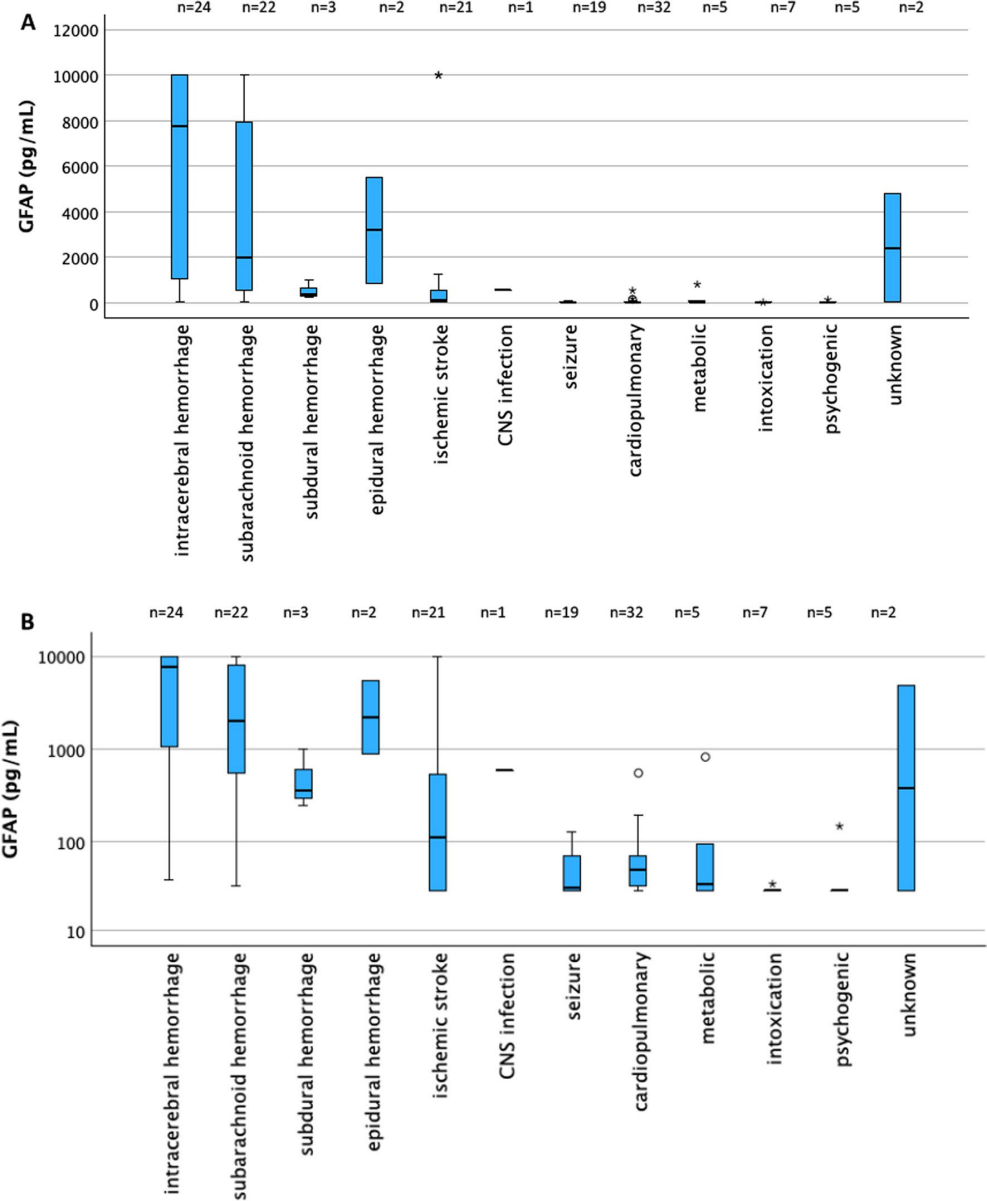
Box plots depicting the distribution of plasma GFAP values according to final diagnosis (i.e., cause of coma). **A** Y axis in metric scale. **B** Y axis in logarithmic scale for better visualization of the smaller GFAP values associated with diagnoses other than intracranial hemorrhage

Figure 3 displays the plasma GFAP concentrations stratified according to diagnosis groups. Median plasma GFAP was 3352 pg/mL (IQR 613–10,001) in patients with intracranial hemorrhage as defined above, and 43 pg/mL (IQR 29–91.25) in patients with other etiologies (*p* < 0.001). Median plasma GFAP was 1214 pg/mL (IQR 147–8764) in patients with a primary cerebral cause of coma (including intracranial hemorrhage as defined above, ischemic stroke and CNS infection, but excluding epileptic seizures), and 35 pg/mL (IQR 29–64.5) in patients with other etiologies (*p* < 0.001). Table 1 depicts the baseline variables and GFAP plasma values stratified according to the final diagnosis. The 5 patients who had their GFAP measurements completed in the prehospital phase showed the following GFAP values and diagnoses: 29 pg/mL (ischemic stroke), 58 pg/mL and 47 pg/mL (cardio-pulmonary event), 32 pg/mL (epileptic seizure), 10,001 pg/mL (intracerebral hemorrhage).

**Table 1:** Baseline characteristics of the study population

	ICH	SAH	SDH	EDH	Ischemic stroke	Seizures	CNS infection	Cardio-pulmonary	Metabolic	Intoxication	Psychogenic	Unknown
n (%)	24 (16.7%)	22 (15.3%)	3 (2.1%)	2 (1.4%)	21 (14.6%)	19 (13.2%)	1 (0.7%)	32 (22.2%)	5 (3.5%)	7 (4.9%)	5 (3.5%)	2 (1.4%)
Female; %	41.7 (10/24)	68.1 (15/22)	33.3 (1/3)	0.0 (0)	57.1 (12/21)	36.8 (7/19)	100.0 (1/1)	28.1 (9/32)	40.0 (2/5)	42.9 (3/7)	20.0 (1/5)	0.0 (0)
Age, mean ± SD; years	77 ± 12	57 ± 21	62 ± 32	63 ± 9	80 ± 12	59 ± 22	66 ± 0	65 ± 17	73 ± 11	44 ± 15	40 ± 20	62 ± 18
GCS, median (IQR)	4 (3–6)	3 (3–5.25)	6 (5.5–14)	4.5 (3.75)	5 (4–7)	4 (3–7)	9	3 (3–4.75)	4 (3–6)	3 (3–9)	6 (3–6)	3
Time span < 2 h (if known) %	37.5 (9/24)	63.6 (14/22)	66.7 (2/3)	100.0 (2/2)	42.8 (9/21)	47.4 (9/19)	0.0 (0)	78.1 (25/32)	0.0 (0)	57.1 (4/7)	40.0 (2/5)	50.0 (1/2)
Anticoagulation; %	50	9	33.3	0	33.3	5.3	0	15.6	40	0	0	50,0
Vitamin K-antagonist	12.5	0	0	0	14.3	0	0	0	0	0	0	0
Dabigatran	0	4,5	0	0	4.8	0	0	0	0	0	0	0
Apixaban	20.8	4.5	0	0	9.5	5.3	0	12.5	20	0	0	0
Rivaroxaban	16.6	0	33.3	0	4.8	0	0	0	0	0	0	50
Edoxaban	0	0	0	0	0	0	0	0	0	0	0	0
Enoxaparin	0	0	0	0	0	0	0	3,1	20	0	0	0
Resuscitation; %	4.2 (1/24)	9.1 (2/22)	33.3 (1/3)	0.0 (0)	0.0 (0)	5.2 (1/19)	0.0 (0)	68.8 (22/32)	40.0 (2/5)	14.2 (1/7)	0.0 (0)	100.0 (2/2)
Neurosurgical Intervention; %	33.3 (8/24)	68.2 (15/22)	33.3 (1/3)	50.0 (1/2)	0.0 (0)	0,0 (0/19)	0.0 (0)	0.0 (0)	0.0 (0)	0.0 (0)	0.0 (0)	0.0 (0)
Trauma; %	16.6 (4/24)	59.1 (13/22)	33.3 (1/3)	100.0 (2/2)	14.3 (3/21)	21.0 (4/19)	0.0 (0)	25.0 (8/32)	0.0 (0)	57.1 (4/7)	20.0 (1/5)	50.0 (1/2)
In-hospital mortality; %	25.0 (6/24)	54.5 (12/22)	66.7 (2/3)	50.0 (1/2)	57.1 (12/21)	10.5 (2/19)	0.0 (0)	43.8 (14/32)	60.0 (3/5)	14.2 (1/7)	0.0 (0)	100.0 (2/2)
GFAP; Median (IQR); pg/mL	7768.5 (983–10,001)	1981.5 (456.25–8113.75)	358 (245)	3192.5 (884)	110 (29–651.5)	31 (29–81)	584 (584)	49 (31.75–70.25)	34 (29–457)	29 (29–29)	29 (29–87.5)	2409.5 (1219.5)

ICH = intracerebral hemorrhage, SAH = subarachnoid hemorrhage, SDH = subdural hemorrhage, EDH = epidural hemorrhage, GCS = Glasgow coma scale

**Fig. 4:**
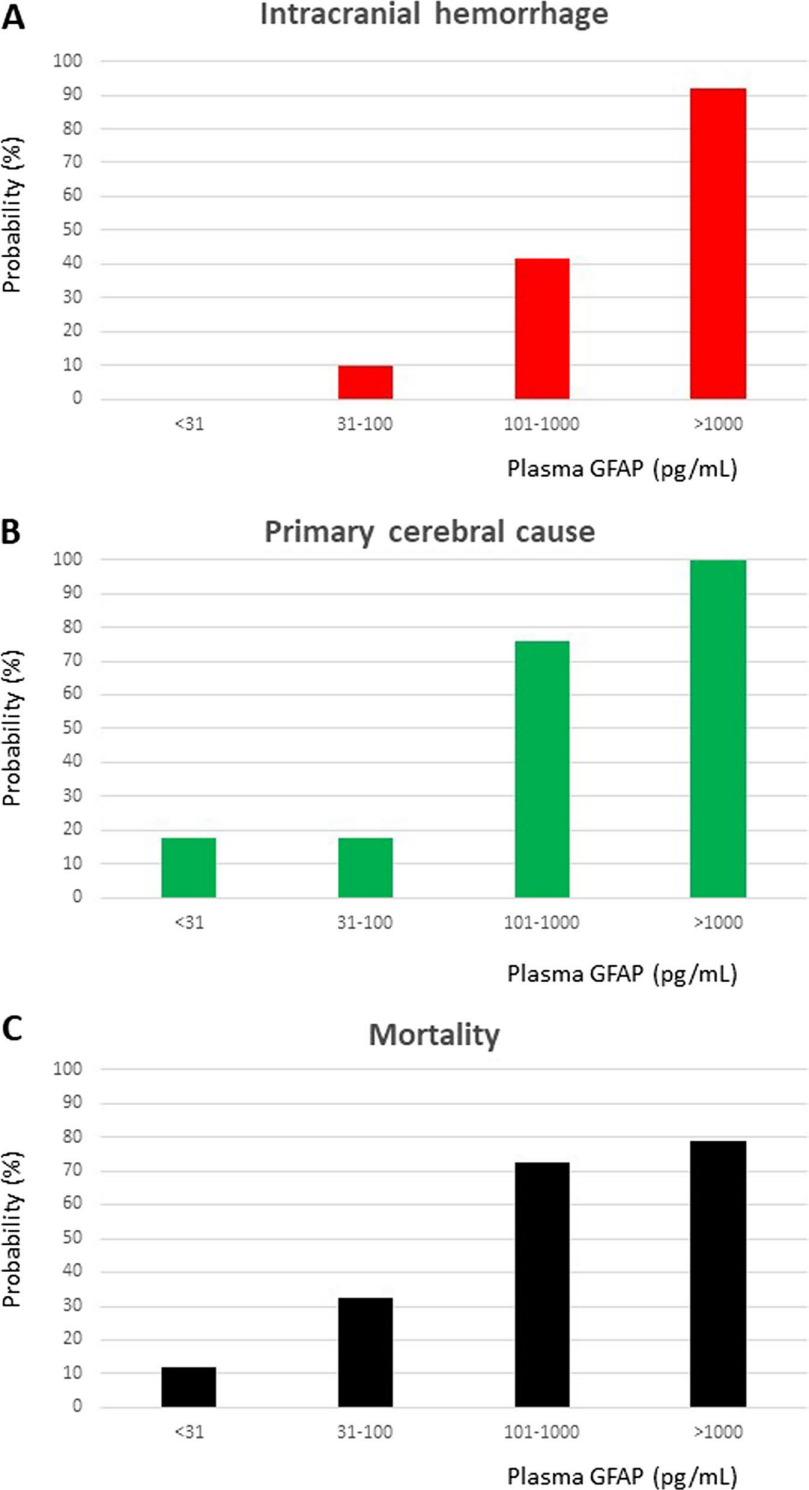
Prehospital probability charts for acute coma patients. Bar charts showing the probability of an intracranial hemorrhage (**A**) and of a primary cerebral cause of coma (**B**) in correlation with GFAP plasma value ranges. In-hospital mortality rates correlated to GFAP plasma value ranges are visualized in graph **C**

The probability of having intracranial hemorrhage increased in parallel with increasing GFAP plasma concentrations (GFAP < 31 pg/mL 0%, GFAP > 1000 pg/mL 92.1%; see Fig. 4). ROC analysis provided an optimal cut-off value of 101 pg/mL for differentiation between intracranial hemorrhage and other coma etiologies (area under the curve 0.925 [95% confidence interval 0.879–0.972], *p* < 0.001). For this cut-off value, sensitivity for identifying intracranial hemorrhage was 94.1% (specificity 78.9%, PPV 71.6%, NPV 95.9%). The probability of having a primary cerebral cause of coma also increased with increasing GFAP plasma concentrations (GFAP < 31 pg/mL 17.6%, GFAP > 1000 pg/mL 100%). When using the same cut-off value of 101 pg/mL (ROC area under the curve 0.883 [0.824–0.942], *p* < 0.001), sensitivity for identifying a primary cerebral cause of coma was 83.6% (specificity 91.2%, PPV 91.0%, NPV 83.8%). In-hospital mortality risk was also strongly associated with prehospital GFAP values as depicted in Fig. 4. When stratifying for age, the diagnostic accuracy was comparable between patients aged above (*n* = 57) and below 75 years (ROC area under the curve 0.931 [0.864–0.998], *p* < 0.001). In addition, patients with very early GFAP measurements after coma onset (< 30 min; *n* = 14) showed a comparable diagnostic accuracy to patients with longer or unknown times from symptom onset to blood withdrawal (ROC area under the curve 1.000 [1.000–1.000], *p* < 0.001).

Among patients with intracranial hemorrhage, 15 out of 51 patients (29%) were on anticoagulation (Table 1). In this group, the median GFAP value was 4475 pg/mL (IQR 906—10,001), and all patients showed GFAP values above 244 pg/mL. The mortality rate among patients with anticoagulation-associated intracranial hemorrhage was 93.3% (14 out of 15 patients).

Patients with ischemic stroke (*n* = 21) showed median plasma GFAP concentrations of 110 pg/mL (IQR 29–651.5). The 3 cases with the highest values had ischemic stroke with hemorrhagic transformation (*n* = 2) and a demarcated hemispheric stroke (*n* = 1) (Table 1).

Prehospital resuscitation was mainly performed in patients with a cardio-pulmonary coma etiology. Overall, 32 patients were resuscitated. Here, a median GFAP concentration of 57 pg/mL (IQR 29.75–89.25) was determined.

## Discussion

Our study demonstrated that prehospital plasma GFAP measurements on a point-of-care platform identify intracranial hemorrhage as the underlying cause of acute coma with high diagnostic accuracy. This allows rapid diagnosis-specific stratification and management of patients by rescue services.

GFAP is a highly brain specific protein that maintains the cytoskeleton of astrocytes. Meanwhile, a substantial body of evidence is available demonstrating that GFAP reliably identifies intracerebral hemorrhage among patients with symptoms of acute stroke (i.e. differentiating intracerebral hemorrhage from ischemic stroke and stroke mimics) [6–10]. Multiple studies confirmed a strong correlation between hematoma volume and GFAP levels in serum or plasma [9, 10]. This suggests that cellular destruction by the expanding hematoma is the most important factor for GFAP release. Similar findings were obtained from studies on patients with TBI. Here, high GFAP levels were particularly associated with traumatic intracerebral bleedings [11, 12]. In contrast, in ischemic stroke, GFAP is released with delay, as necrosis in ischemic stroke usually does not occur within the first hours after symptom onset [22].

Our study confirmed that any type of intracranial hemorrhage causes significant GFAP release. Of course, in patients with acute coma, the volume of the intracranial bleedings is on average larger than in patients having only mild stroke symptoms. This increases the diagnostic accuracy of GFAP for this indication (i.e. increasing the sensitivity for detecting intracranial hemorrhage). Due to a lack of relevant astroglial destruction in short time, other causes of acute coma are not associated with GFAP release, including epileptic seizures, intoxication, metabolic derangement, and cardio-pulmonary events. However, it is expected that in case of prolonged exposure of brain cells to such pathological conditions (e.g. hypoxia due to pulmonary embolism or severe hypoglycemia) GFAP levels increase over time, along with subsequent (but not rapid) glial necrosis [23, 24]. The same is true for ischemic stroke as a primary cause of acute coma (e.g., “top of the basilar syndrome”). In early time frames, GFAP levels are low (“potentially salvageable tissue”), whereas in prolonged time frames GFAP increases in parallel with the occurrence of ischemic necrosis. GFAP may also increase in ischemic stroke associated with hemorrhagic transformation [6–10]. In consequence, GFAP testing do not allow a reliable identification of ischemic stroke as the underlying coma etiology. Vice versa, elevated values are not exclusively associated with intracranial hemorrhage.

Our study is the first to describe a blood-based measure for prehospital identification of intracranial hemorrhage in patients with acute coma. In view of the fact that the point-of-care device used here (especially the former model i-STAT 1® of this device) is widely available and routinely applied for diagnostics in the prehospital phase (e.g., for blood gas analysis), our findings could have major impact on triage and management of these critically ill patients on short notice [25–28]. Specifically, the probability indicator charts as illustrated in Fig. 4 can be used as easy-to-read “pocket cards” to estimate the probability of an intracranial hemorrhage or a primarily cerebral cause of coma, respectively. GFAP values above 1000 pg/mL were associated with intracranial hemorrhage or another primary cerebral cause of coma in all cases, allowing reliable biomarker-based triage of patients with low error rate. Our study was performed in a highly-populated metropolitan area in Germany with short distances to the tertiary care hospital. Nevertheless, the pre-hospital and intra-hospital procedural times to assess and treat comatose patients are still significant. Not before stabilization (including intubation in selected cases) patients can be transported, opening a benefit of GFAP testing in this setting. Of course, this test may unfold its full potential in remote and resource-limited areas, where any procedural improvement may directly translate into a better prognosis of patients [29–33].

The high PPV in patients with increased GFAP values opens the door to an earlier treatment of patients with severe intracranial hemorrhage. This includes rapid blood pressure lowering as well as the reversal of anticoagulation (e.g. by Andexanet alpha or Idarucizumab) in the prehospital phase [34, 35]. The particularly high mortality rate in anticoagulated coma patients with intracranial hemorrhage reveals the urgent need for early initiation of therapeutic measures in order to reduce bleeding sequelae. Occasionally, acute coma patients with suspected cardio-pulmonary events receive anticoagulants which could induce clinical worsening in case of (co-incidental) intracranial hemorrhage [36, 37]. A GFAP test prior to any prehospital anticoagulation administration could help to minimize fatal consequences resulting from undiagnosed intracranial hemorrhage.

At present a limitation of our study is that the point-of-care test requires plasma to fill the GFAP test cartridges. For most of the included patients in this study, blood was taken in the prehospital phase and centrifuged in our routine laboratory shortly after arrival. In a small subset of patients, however, we were able to prove that prehospital GFAP measurements are feasible, even if a centrifugation step is necessary. In the future, a GFAP full blood point-of-care test may substantially reduce procedural efforts.

## Conclusions

In summary, increased GFAP plasma values in patients with acute coma identify intracranial hemorrhage with high diagnostic accuracy. Prehospital GFAP measurements on a point-of-care platform may allow rapid stratification according to the underlying cause of coma by rescue services. This could have major impact on triage and management of these critically ill patients.

## Data Availability

The datasets used and analyzed during the current study are available from the corresponding author upon reasonable request.

## Abbreviations

GCS: Glasgow Coma Scale
GFAP: Glial fibrillary acidic protein
UCH-L1: Ubiquitin C-terminal hydrolase L1
TBI: Traumatic brain injury
CNS: Central nervous system
FDA: Food and Drug Administration
CT: Computed Tomography
IQR: Interquartile range
ROC: Receiver operating characteristics
PPV: Positive predictive value
NPV: Negative predictive value
min: Minutes
ICH: Intracerebral hemorrhage
SAH: Subarachnoid hemorrhage
SDH: Subdural hemorrhage
EDH: Epidural hemorrhage
